# Polarity-active NIR probes with strong two-photon absorption and ultrahigh binding affinity of insulin amyloid fibrils†

**DOI:** 10.1039/d0sc03907a

**Published:** 2021-01-05

**Authors:** Li Li, Zheng Lv, Zhongwei Man, Zhenzhen Xu, YuLing Wei, Hua Geng, Hongbing Fu

**Affiliations:** Beijing Key Laboratory for Optical Materials and Photonic Devices, Capital Normal University Beijing 100048 China xuzhenzhen@cnu.edu.cn hbfu@cnu.edu.cn; Key Laboratory of Molecular Optoelectronic Sciences, Institute of Molecular Plus, Tianjin Collaborative Innovation Center of Chemical Science and Engineering, Tianjin University Tianjin 300072 China

## Abstract

Amyloid fibrils are associated with many neurodegenerative diseases. *In situ* and *in vivo* visualization of amyloid fibrils is important for medical diagnostics and requires fluorescent probes with both excitation and emission wavelengths in the far-red and NIR region, and simultaneously with high binding-affinity to amyloid fibrils and the ability to cross the blood–brain barrier, which, however, remain a challenge. Here, we rationally design and synthesize an excellent polarity-sensitive two-photon excited NIR fluorophore (TZPI) based on a donor (D)–acceptor (A)-ion compound. The electron-rich carbazole group and the ionic pyridinium bromide group, linked by an electron-poor π-conjugated benzothiadiazole group, ensure strong near infrared (NIR) emission. Furthermore, the lipophilic carbazole together with the benzothiadiazole group facilitates docking of the probe in the hydrophobic domains of amyloid aggregates with the dissociation constant *K*_d_ = 20 nM and 13.5-fold higher binding affinity to insulin fibrils than the commercial probe ThT. On association with the amyloid fibrils, the tiny decrease in polarity leads to a large increase in its NIR emission intensity with an on–off ratio > 10; meanwhile, the TZPI probe exhibits a quantum yield of up to 30% and two-photon absorption cross-section values of up to 467.6 GM at 890 nm. Moreover, the application of TZPI in two-photon imaging is investigated. The ultrahigh binding affinity, the strong NIR emission, the good two-photon absorption properties, the high photo-stability, the appropriate molecular mass of 569 Da and the lipophilicity with log *P* = 1.66 ± 0.1 to cross the BBB make TZPI promising as an ideal candidate for detecting amyloid plaques *in vivo*.

## Abstract 2

A polarity-active NIR probe based on the transformation from the CT state to the LE state for two-photon imaging of amyloid fibrils.

## Graphical abstract

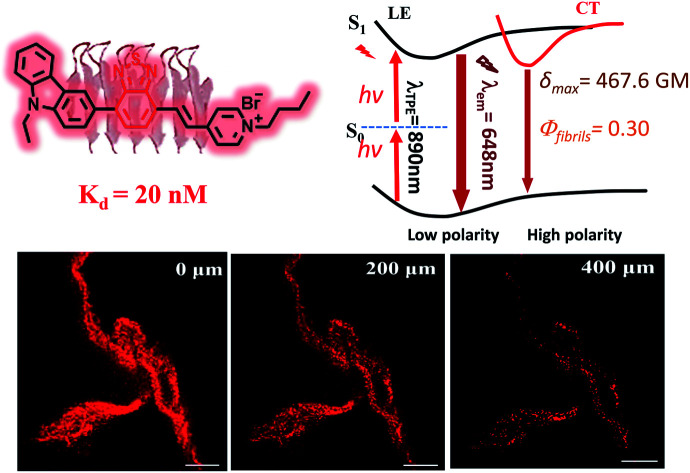

## Introduction

Amyloidal fibrils are the denatured fibrous form of protein aggregates, and their excessive accumulation in organs and tissues can lead to biological dysfunction and pathological symptoms, such as Alzheimer's and Parkinson's diseases, type II diabetes and prion diseases.^1–3^ Therefore, both the sensitive detection and imaging of the protein plaques and tangles and the mechanistic understanding of the amyloid deposition processes in molecular detail are of diagnostic urgency. Note that the fibril formation is not only limited to proteins related to neurodegenerative diseases, but is an intrinsic property of the polypeptide backbones. Insulin is a protein that readily undergoes the fibrillogenesis process and has been an excellent model for studying protein amyloidogenesis. Over the past decades, many techniques for detecting amyloid fibrils have been developed, including magnetic resonance imaging (MRI),^4,5^ positron emission tomography (PET),^6^ single photon emission computed tomography (SPECT)^7,8^ and fluorescence microscopy. Among them, fluorescence microscopy has attracted increasing interest, benefitting from several advantages, including lower cost, no exposure to radioactivity, availability and ease of operation, high sensitivity and friendliness to biological samples.

**Fig. 1:**
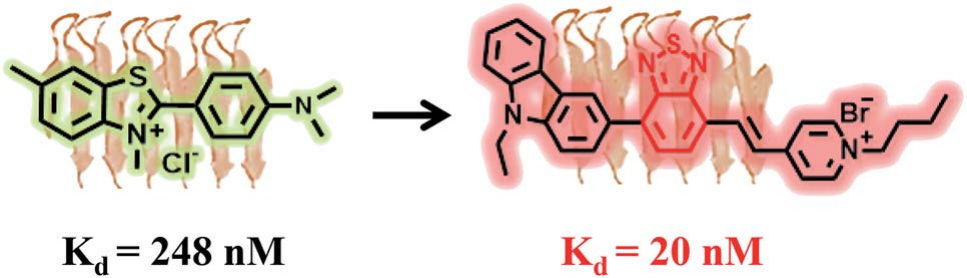
Rational design of the molecule TZPI with a binding affinity to insulin fibrils 13.5-fold stronger than that of commercially available ThT.

Thioflavin T (ThT) is a gold standard fluorescence marker of amyloid fibrils with a relatively high binding affinity with *K*_d_ = 270 nM (Fig. 1) that has been widely used for *in vitro* biomedical assays. When binding to the surface of the β-sheet of amyloid fibrils mainly through hydrophobic interaction, the restriction of intramolecular motion prohibits nonradiative decay and leads to a more than 100-fold fluorescence enhancement with a maximum emission at 485 nm.^9–11^

In general, an ideal fluorescent probe should have the following properties: (i) appropriate molecular mass (<600 Da) and lipophilicity (log *P* value between 1 and 3) to cross the blood–brain barrier (BBB), (ii) high binding affinity and specificity to amyloid fibrils, and (iii) a significant change in fluorescence properties upon binding to amyloid fibrils.

Compared to visible light, far-red and near-infrared (NIR) irradiation (650–1000 nm) offer several advantages, such as better separation from biosubstrate autofluorescence, low light-scattering and deep tissue-penetration, and minimal photo-damage to living organisms. Thus, fluorescent probes with both excitation and emission wavelengths in the far-red and NIR region, in addition to high binding-affinity to amyloid fibrils and ability to cross the BBB are ideal candidates for *in situ* detection and visualization of amyloid fibrils.^12–16^ Chongzhao Ran *et al.* synthesized a series of difluoroborate diketone compound-based NIR probes for detection of amyloid deposits.^17–19^ Upon interacting with amyloid aggregates, the probe CRANAD-2 exhibits a 70-fold intensity increase at a maximum emission of 715 nm with *λ*_max(ex)_ = 640 nm and a high affinity for amyloid aggregates with *K*_d_ = 38.0 nM, which meets the requirements of a NIR probe for detecting amyloid deposits noninvasively *in vivo*.^18^

Herein, a new D–A-ion polarity-active probe (*E*)-1-butyl-4-(2-(7-(9-ethyl-9*H*-carbazol-3-yl)benzo[*c*][1,2,5]thiadiazol-4-yl)vinyl)pyridin-1-ium bromide (TZPI) with both excitation and emission in the far-red and NIR region for amyloid fibrils was designed and synthesized. The electron-rich carbazole group and the ionic pyridinium bromide group, linked by an electron-poor π-conjugated benzothiadiazole group, ensure strong near infrared (NIR) emission with the maximum emission wavelength around 650 nm. After binding with amyloid plaques, the small polarity decrease from water to the microenvironment of amyloid fibrils leads to a 15-fold emission intensity enhancement from 0.02 to 0.3. Furthermore, the lipophilic carbazole together with the benzothiadiazole group facilitates docking of the probe in the hydrophobic domains of amyloid aggregates with 13.5-fold higher binding affinity of insulin fibrils with *K*_d_ = 20 nM than that of the commercial probe ThT, as shown in Fig. 1. Due to the strong intramolecular charge transfer (ICT) between the donor and acceptor, the TZPI exhibits relatively strong two-photon absorption cross-section values of up to 467.6 GM in the excitation range of 820–930 nm. Together with the excellent photo-stability and the appropriate molecular mass and lipophilicity to cross the BBB, the TZPI is promising as an efficient polarity-active NIR probe in two-photon fluorescence imaging of amyloid fibrils.

## Results and discussion

Increasing efforts have been made in the search for novel NIR probes for detection of amyloid deposits.^12–20^ Among them, high binding affinity to amyloid aggregates is a crucial property. Recently, we reported the rational design of a new class of near-infrared (NIR) probe, CTPB, for amyloid fibrils based on the polarity-activated fluorescence turn-on mechanism.^20^ The carbazole-pyridinium bromide building blocks linked by an electron-rich π-conjugated thiophene-bridge ensure strong NIR emission. Binding to a relatively non-polar microenvironment of amyloids fibrils leads to a 10-fold increase in emission. Moreover, the CTPB has the same binding mode as ThT with a comparable binding affinity with a *K*_d_ about 235 nM. In this work, to achieve better binding affinity and ensure both the excitation and emission in the NIR region, we designed a new polarity active NIR probe based on a new D–A-ion structure with a molecular weight of 569 Da. We introduce the electron-acceptor (A) unit benzothiadiazole instead of the thiophene group as a specific marker group to improve the binding ability with fibrils. Furthermore, the lipophilic electron-donor (D) unit carbazole was introduced as a π-conjugated backbone for achieving NIR wavelength emission and two-photon absorption and producing a fluorescence quantum yield which is sensitive to the polarity of the surrounding environment. Meanwhile, the pyridinium bromide group was introduced to increase its water solubility in case of unnecessary aggregation before binding to amyloid aggregates.^21,22^ In addition, the lipophilicity value (log *P*) of TZPI was theoretically predicted to be 1.66 in Fig. S1† based on the molecular fragment calculation method.^23^ Furthermore, the experimentally measured partition coefficient in *n*-octanol^17^ is 1.56 in Fig. S2,† which is basically in line with the theoretical value. The lipophilicity with log *P* = 1.66 ± 0.1 was a well-matched value with the estimated range (1 and 3) for penetrating the BBB. Further investigations into the BBB permeability of our TZPI probe are underway.

**Fig. 2:**
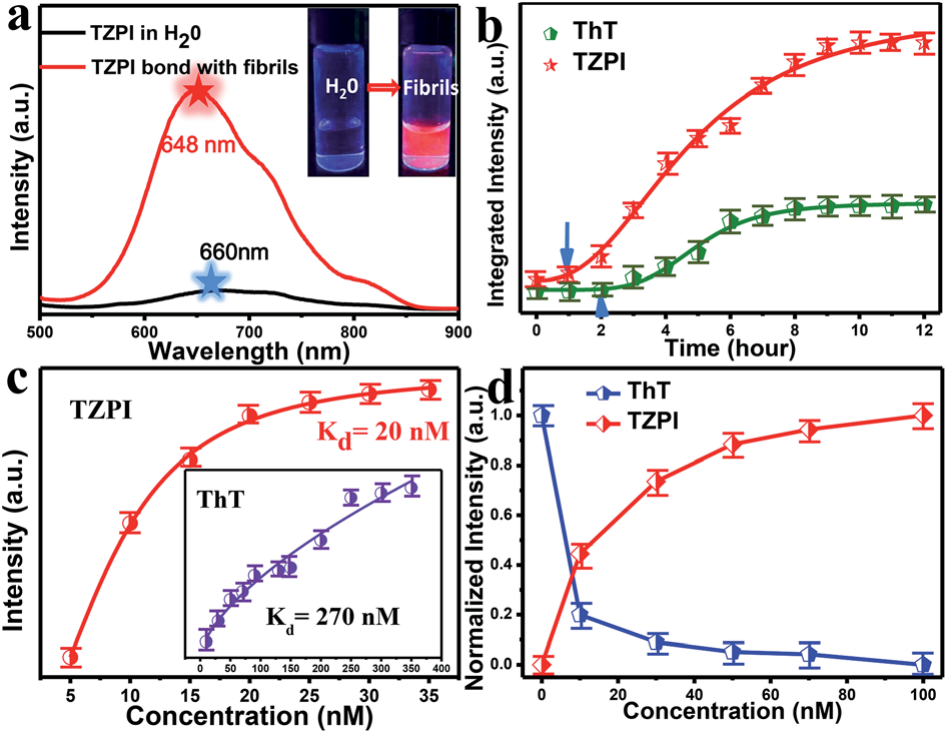
(a) Emission spectra of TZPI (20 nM) dissolved in Tris–HCl buffer water solution (black) and mature fibril solution (red), respectively. *λ*_ex_ = 480 nm. Photographs of mixtures of TZPI with water and fibrillary forms of insulin were obtained under UV illumination. (b) Integrated intensity of TZPI (red, 20 nM) and ThT (green, 20 nM) monitoring insulin (10 μg mL^−1^) fibrillation, *λ*_ex_ = 480 nm for TZPI, 410 nm for ThT. (c) The saturation binding of TZPI to the fibrillary form of insulin. The dissociation constants *K*_d_ of TZPI were fitted using a one site binding equation. The resulting *K*_d_ for TZPI binding with fibrils is 20 nM. Inset: the dissociation constant (*K*_d_) of ThT is 270 nM. (d) TZPI (0–100 nM) effectively displaces ThT from the ThT/fibril insulin aggregate complex.

As depicted in Schemes S1 and S2,† the molecule TZPI was originally synthesized by a two-step reaction. The structure was confirmed by ^1^H NMR and mass spectrometry (Fig. S3–S6†). Fig. S7† shows the absorption and fluorescence emission spectra of TZPI in water with a concentration of 20 nM. The absorption peak of TZPI in water is at 462 nm with a molar extinction coefficient of 51 380 M^−1^·cm^−1^. Note that TZPI exhibits a high solubility in water, up to 4 μM as determined by the UV-vis titration method in Fig. S8.† The fluorescence emission peak of TZPI in Tris–HCl buffer solution is centred at 660 nm with the tail extending beyond 750 nm as shown in Fig. 2a, falling in the best range for NIR fluorescence probes.

In this work, bovine insulin was chosen as a model to investigate misfolding and aggregation of protein. Insulin amyloid fibrils were prepared by dissolving the protein in water solution (0.01 M HCl, 5 mg mL^−1^ for insulin) and incubated in an orbital thermomixer with constant agitation at 600 rpm at 60 °C for 24 h. Then the fibrils were characterized by transmission electron microscopy (TEM) as shown in Fig. S9a.† Upon addition of incubated amyloid fibrils, a significant emission intensity (15-fold) enhancement appears as shown in the inset of Fig. 2a. Meanwhile, the interaction of TZPI with the insulin amyloid fibrils leads to a bathochromic shift of the emission maximum from 660 to 648 nm and the absorption maximum from 462 nm to 460 nm. Several reports proposed that the conversion of protein monomers to aggregates is accompanied by a difference in the local microenvironment polarity.^24–29^ Thus, the bathochromic shift in the absorption and emission spectra of TZPI may arise due to the reduced polarity at the TZPI binding site in the amyloid fibrils.

To make sure that TZPI is binding specifically to insulin fibrils and not to native protein, we compared the absorption and fluorescence spectra of TZPI containing native bovine insulin with those of TZPI in Tris–HCl water solution. Note that the TZPI in Tris–HCl solution remains weakly emissive after the addition of native bovine insulin and no difference is found as shown in Fig. S10,† suggesting that TZPI molecules do not interact with native bovine insulin.

The distinct emission behaviours of TZPI in the presence of native and fibrillary forms of insulin, especially the turn-on NIR emission, prompt us to explore the possibility of utilizing it to monitor the kinetic process of amyloid fibrosis. Using TZPI and ThT as *ex situ* probes as shown in Scheme S3a,† an aliquot of the insulin solution taken out from the incubation mixture at a defined time was diluted with Tris–HCl buffer to pH ≈ 7 which was consistent with physiologically relevant conditions, followed by the addition of the probes. The concentration of both TZPI and ThT is 20 nM. The maximum emission intensity of the TZPI and ThT was utilized. The two S-curves including an initial lag phase, an exponential growth phase and a final plateau phase in Fig. 2b well describe the amyloid aggregation process which involves three distinct steps: (I) nucleation, (II) elongation, and (III) equilibration. Compared with ThT, TZPI presents a stronger emission during this process benefitting from a higher fluorescence quantum yield of TZPI (*Φ* = 0.30). Besides, the response time of TZPI to nucleating proteins is significantly 1–2 h earlier than that of ThT, indicating that TZPI might have higher binding affinity which can detect trace aggregated fibrils in the early incubation period.

In order to verify whether TZPI has an effect on the process of fibrosis aggregation, the study using TZPI as an *in situ* probe was performed as shown in Scheme S3b and Fig. S11.† Compared with the study using TZPI as an *ex situ* probe as shown in Fig. 2b, the fluorescence intensity, the inflection point and the plateau period of the two S-curves were basically consistent. Besides, Fig. S9† shows that the morphology of the mature fibrils formed using TZPI as an *in situ* probe shows almost no difference in comparison to that without adding any probe molecules. Then we measured the kinetic parameters using TZPI as an *in situ* or an *ex situ* probe with varying concentrations of insulin (0.87–2.62 μM) and a constant concentration of TZPI (20 nM). As shown in Fig. S12a and d,† all the fluorescence data obtained can describe the kinetic process of fibrosis perfectly. Theoretical analysis of aggregation kinetics was performed using the software AmyloFit.^30^ Fig. S12b and e† show that the scaling exponents from a log–log plot of half time against initial peptide concentration are −1.17 and −1.21, respectively. The values are almost equal and both consistent with a dominant secondary nucleation pathway characterized by a monomer dependence of *n*_2_ = 2 and a contribution from primary nucleation with a reaction order of *n*_c_ = 2. Furthermore, the data we fit based on the dominant secondary nucleation pathway are consistent with the experimental data over the full reaction time course (Fig. S12c and f†).^31^ All the above experimental results indicate that TZPI does not have an effect on the fibrillation kinetics.

High binding affinity is another crucial property of the amyloid fibril probe. To quantitatively evaluate the binding affinities of the aggregated amyloid fibrils, the *K*_d_ for TZPI/fibrils was derived by a fluorescence titration method and the value was found to be 20 nM as shown in Fig. 2c and S13.† Significantly, the binding affinity of TZPI was 13.5-fold higher than that of ThT with *K*_d_ = 270 nM. Moreover, compared to the CTPB probe,^19^ simply replacing the thiophene with benzothiadiazole leads to 11-fold higher binding affinity. These results revealed that TZPI is a superior probe for sensitive binding to fibrils. The higher binding affinity can be attributed to the stronger interaction between TZPI and fibrils. It is reported that the formation of fibrils involves interplay of hydrophobic and electrostatic interactions and the occurrence of a cross β-structure.^9–11^

**Fig. 3:**
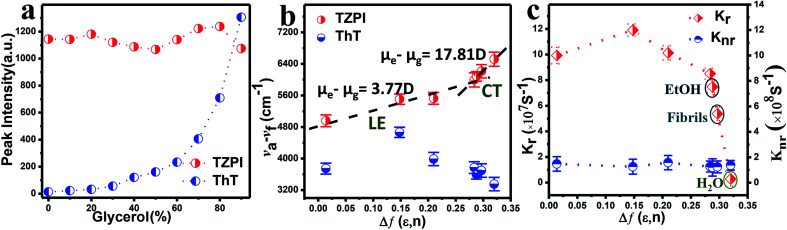
(a) Plot of the emission intensity for each solution *versus* percentage glycerol present. (b) Linear correlation of the orientation polarization (Δ*f*) of the solvent media with the (*ν*_a_ − *ν*_f_) for TZPI and ThT. The lines are in the low- and high-polarity regions (LE and CT), respectively. (c) The orientation polarization (Δ*f*) of the solvent media with the *k*_nr_ and *k*_r_ for TZPI in different solvents.

Furthermore, we can see from Fig. S14 and S15† that fluorescence intensity changes little over a large span of salt concentrations or the pH range, indicating that there is no significant electrostatic interaction between TZPI and the fibrils. This excludes the electrostatic interaction as the major driving force for TZPI binding with insulin amyloid fibrils. We then add TZPI to ThT-bound fibrillary aggregates. As shown in Fig. 2d, a remarkable decline in the fluorescence intensity of ThT was observed at 485 nm and the intensity of the NIR emission peak located at 648 nm increased dramatically. Besides, the removal efficiency of TZPI to ThT highly reaches 97.5%. The results indicate that TZPI may compete for the same binding sites with ThT and show higher binding affinity with amyloid fibrils than ThT. It is reported that the binding location of ThT for the strongly emissive population predominantly involves hydrophobic interaction through the π–π interaction between the dimethylaniline moiety and the peptide backbone and the CH–π interaction with the amino acid residues.^9,10^ Typical of ICT compounds, the molecule TZPI is polarity-active. Together with the blue-shifted emission of TZPI, these results all indicate that TZPI binds to the hydrophobic pockets of aggregated amyloid fibrils^32,33^ and the lipophilic carbazole together with the benzothiadiazole group has a higher hydrophobic interaction than that of ThT. It has been reported that when ThT entered into the hydrophobic pockets and bound to the fibrils, the conformational freedom and rational restriction of ThT decreased, resulting in the enhancement of fluorescence.^9–11^ Therefore, there might be two mechanisms that can be proposed to account for the turn-on effect of TZPI binding to amyloid fibrils: (i) the change of the polarity of the microenvironment from water to the hydrophobic domains of amyloid fibrils and (ii) the restriction of the torsional motion of vinyl groups. To verify this, we changed the viscosity of the system by adjusting the ratio of ethanol to glycerol. The experimental results in Fig. 3a and S16† show that the fluorescence intensity of ThT increased significantly with the increase of the glycerol content in the mixed system (about 100 times), while the emission intensity values of TZPI are almost constant, so we exclude the effect of restriction and maintain that the change of polarity in the environment is the dominant factor for the enhancement of fluorescence brightness.

We then investigated the polarity-active mechanism of the significant fluorescence enhancement after TZPI binding to the fibrils. The optical properties of TZPI are investigated as a function of the solvent polarity. As shown in Fig. S17 and Table S1,† the emission maximum (*λ*_f_) was gradually red-shifted with the increasing solvent polarity from a dielectric constant (*ε*) of toluene (*ε* = 2.38, *λ*_f_ = 606 nm) to water (*ε* = 80.4, *λ*_f_ = 660 nm). Based on the Lippert–Mataga equation, the difference between the excited- and ground-state dipole-moments, *μ*_e_ − *μ*_g_ of TZPI can be determined according to the slope of Stokes displacement (*ν*_a_ − *ν*_f_) relative to the orientation polarization (Δ*f*). As shown in Fig. 3b, the plot shows two linear regions, which indicates the presence of two different excited states in low and high polarity solvents. In the high-polarity solvents, the *μ*_e_ − *μ*_g_ was calculated to be 17.81 D and was attributed to the CT states with a low emission intensity of *φ*_FL_ = 0.02 in water. In the low-polarity range from Δ*f* = 0.014 (toluene) to 0.288 (ethanol), the corresponding *μ*_e_ − *μ*_g_ of TZPI decreased to 3.77 D owing to the LE states with a high emission intensity of *φ*_FL_ = 0.39 in ethanol (Table S1†). Moreover, the (*ν*_a_ − *ν*_f_) of the TZPI binding to amyloid fibrils falls in the polarity with Δ*f* = 0.297 and with *φ*_FL_ = 0.30, which is near the polarity of ethanol and consistent with previous reports,^33^ while for ThT (blue dot in Fig. 3b and Table S2†), the relationship of the (*ν*_a_ − *ν*_f_) relative to *f* does not show similar transition properties.

To gain insight into the actual mechanism of the turn-on behaviour, we performed time-resolved fluorescence measurements of TZPI in different solvents. Moreover, the radiative decay rates (*k*_r_) and the nonradiative decay rates (*k*_nr_) were calculated based on the equations *k*_r_ = *Φ*/*τ* and *Φ* = *k*_r_/(*k*_r_ + *k*_nr_).^34^ In Fig. 3c and Table S1,† we can see that from Δ*f* = 0.288 (ethanol) to 0.320 (water), TZPI presented a single-exponential PL decay process, yielding decreased radiative rates from 7.48 × 10^7^ s^−1^ to 0.29 × 10^7^ s^−1^ and almost the same nonradiative decay rates of 1.20–1.58 × 10^8^ s^−1^, respectively, which are the typical characteristics of CT state luminescence. Moreover, the *k*_r_ and *k*_nr_ of the TZPI binding to amyloid fibres are 5.38 × 10^7^ s^−1^ and 1.24 × 10^8^ s^−1^, which match with the data of the polarity with Δ*f* = 0.297. This phenomenon is completely different from that of ThT, the *k*_nr_ of which decreases rapidly because of the restriction of intramolecular motion (RIM) when binding to the amyloid fibrils.^35,36^ In summary, unlike the RIM-activated turn-on mechanism of ThT, the emission enhancement of TZPI before and after binding with fibrils can be ascribed to the transition from a CT state to a LE state, leading to the desirable on–off characteristics during the binding to amyloid aggregates.

**Fig. 4:**
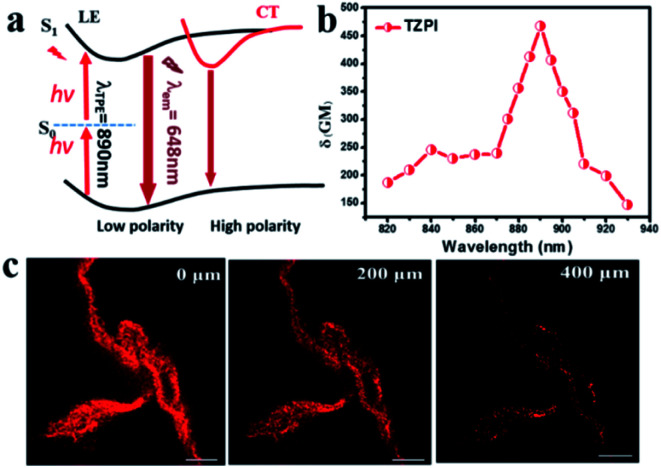
(a) The scheme of two-photon excited NIR fluoresce of the TZPI probe. Here S_0_: ground state, S_1_: first excited singlet state. (b) Calculated TPA cross-section of TZPI binding to amyloid fibres. (c) Two-photon scanning luminescence images of amyloid protein structures at the equilibration phase.

The strong push–pull dipolar character of the D–A-ion type molecular structures can improve the two-photon absorption (TPA) cross section effectively.^37^ As shown in Fig. 4a, upon near-IR laser excitation (820–930 nm), the TZPI molecule can absorb the pumping energy *via* a TPA process to the S_1_ state. The change in polarity before and after TZPI binding with amyloid plaques leads to a transition from the weakly emissive CT state to the highly emissive LE state, giving rise to a NIR fluorescence turn-on probe. The TPA cross section (*δ*_R_) of TZPI binding to amyloid fibres was measured through the two-photon-excited fluorescence method.^38^ We measured TPF using a solution of Rhodamine B (*δ*_S_) in ethanol as a reference, calculated with the equation, *δ*_R_ = *δ*_S_(*F*_*S*_*Φ*_R_*C*_R_*n*_s_/*F*_R_*Φ*_S_*C*_S_*n*_R_), where *F* represents the intensity of two photon excited fluoresce (TPF), *C* is the concentration, and *n* denotes the refractive index of the solvent. As shown in Fig. 4b, TZPI displays excellent two-photon absorption activity in the range of 820–930 nm and exhibits a maximum two-photon absorption cross-section *δ*_R_ of 467.6 GM at 890 nm, which is about 2-fold larger than that of the commercial dye Rhodamine B which is *δ*_S_ = 208.74 GM at 840 nm.^39^

To exclude the contribution of a photon process, we investigated the relationship between the peak intensity of the two-photon excited fluorescence spectra of TZPI in ethanol solution and the laser intensity at 890 nm. The log–log plot showed that the slope was 1.88, which validated the two-photon nature of the absorptions as shown in Fig. S18.†

The photo-stability of TZPI was studied under continuous laser excitation as shown in Fig. S19.† Compared with Rhodamine B, the TZPI in ethanol solution exhibited excellent photo-stability without an observable decrease in fluorescence intensity after irradiation at 800 nm for 4000 s.

Considering the excellent two-photon activity and the photo-stability, the application of two-photon imaging was investigated using a commercial microscope TCS SP5 MP FCS, Leica Instruments. To explore the potential use of the TPZI for deep tissue TPF imaging, we investigated the imaging depth of TPZI in a turbid tissue phantom. Intralipid was chosen as a mock tissue because the scattering properties of the simulative tissues were similar to those of real tissue.^40^ As shown in the schematic of our imaging setup (Scheme S1†), the thickness between the two pieces of coverslips was adjusted by placing double-sided adhesive with different layers. And 1% intralipid solution was injected into the tunable gap between the two coverslips to form the simulative tissue. The scattering properties of the simulative tissues were similar to those of real tissue. A drop of the TZPI/fibril solution was dripped on the top coverslip. And an inverted two-photon microscope with a 20× long working objective lens (0.45 NA) was used to image the amyloid fibrils at different depths in the mock tissue. As shown in Fig. 4c, intense NIR luminescence and the clear fibrillary structures were always observed with depths ranging from 0 to 400 μm in the tissue phantom under excitation with femtosecond laser pulses at 890 nm. Even at a depth of 400 μm, the fibrils bound with TZPI probes can be imaged with high resolution and a high signal-to-noise ratio. Obviously, TZPI is a perfect amyloid fibril probe for two-photon imaging with high photostability and deep tissue-penetration. In addition, we carry out the experiment of TZPI binding with the Aβ fibrils, which is the major component of amyloid plaques in the brains of individuals affected by Alzheimer's disease (AD). A significant emission intensity (14-fold) enhancement of TZPI with a *K*_d_ = 58 nM appears upon addition of incubated Aβ_42_ fibrils, indicating a high on–off ratio > 10 and a high binding affinity. All results together with the appropriate molecular mass and lipophilicity to cross the BBB reveal TZPI as a potential two-photon imaging probe for achieving NIR-to-NIR imaging of brain amyloid plaques in real-time and *in situ* visualization. Further investigations are underway.

## Conclusions

In general, we have developed a polarity-active probe TZPI for amyloid fibrils with both excitation and emission in the far-red and NIR region based on a simple D–A-ion structure. With a tiny change from water (Δ*f* = 0.320) to amyloid fibrils (Δ*f* = 0.297), a 15-fold emission intensity enhancement (from 0.02 to 0.3) was realized. Moreover, the TZPI probe exhibits ultrahigh binding affinity (*K*_d_ = 20 nM) which is 13.5-fold higher than that of the commercial probe ThT. As a TPF probe, TZPI exhibits a large TPF cross-section of 467.6 GM at 890 nm and a high photostability. Moreover, the application of TZPI in two-photon imaging was investigated. The ultrahigh binding affinity, the strong NIR emission, the good two-photon absorption properties, the high photo-stability, the appropriate molecular mass and the lipophilicity to cross the BBB make TZPI promising as an ideal candidate for detecting amyloid fibrils *in vivo*.

## Conflicts of interest

There are no conflicts to declare.

## Supplementary Material

SC-012-D0SC03907A-s001
