# Supplementary material for: Polarity-active NIR probes with strong two-photon absorption and ultrahigh binding affinity of insulin amyloid fibrils
Source: Chem Sci. 2021 Jan 5;12(9):3308–13. doi: 10.1039/d0sc03907a (PMC8179388; doi:10.1039/d0sc03907a)
Supplement: SC-012-D0SC03907A-s001 [file SC-012-D0SC03907A-s001.pdf]

Electronic Supplementary Material (ESI) for Chemical Science.
This journal is © The Royal Society of Chemistry 2021

Supplementary Information

Polarity-active NIR probes with strong two-photon absorption and ultrahigh binding affinity of insulin amyloid fibrils

Li Li,[a] Zheng Lv,[a,b] Zhongwei Man,[a] Zhenzhen Xu,*[a] YuLing Wei,[a] Hua Geng,[a] and Hongbing Fu*[a, b]

[a.] Beijing Key Laboratory for Optical Materials and Photonic Devices, Capital Normal University, Beijing 100048, China.

[b.] Tianjin Key Laboratory of Molecular Optoelectronic Sciences, Institute of Molecular Plus, Tianjin Collaborative Innovation Center of Chemical Science and Engineering, Tianjin University, Tianjin 300072. China.

Correspondence and requests for materials should be addressed to Z.Z.X. (email: xuzhenzhen@cnu.edu.cn), or to H.B.F. (email: hbfu@cnu.edu).

Materials and Methods

Materials and reagents

All reagents for organic synthesis were purchased from Alfa Aesar and used as received without further purification. Other organic solvents (HPLC grade) were purchased from Beijing Chemical Agent Ltd., China. Ultrapure water with a resistivity of 18.2 MΩ cm^{-1}, produced using a Milli-Q apparatus (Millipore), was used in all experiments. TZPI was synthesized and confirmed by mass spectrometry (GCT-MS Micromass, UK) and ^{1}H NMR. Thioflavin T (ThT) and bovine insulin were purchased from Solarbio with no further purification.

The method of measuring log P

1. **Theoretical determination of log P based on the molecular fragment calculation method.**

The log P value we mentioned was acquired according to the method in the previous literature.[1] First, we draw the target molecule TZPI using ACD/ ChemSketch™ and generate the appropriate SMILES notation (Fig. S1). Then we copy the resulted SMILES notation of analyzed compound and paste it into the correct bar of the main page of ALOGPS program (http://www.vcclab.org/lab/alogps/), then click the icon "submit". After that we get the exact value of log P is 1.66.

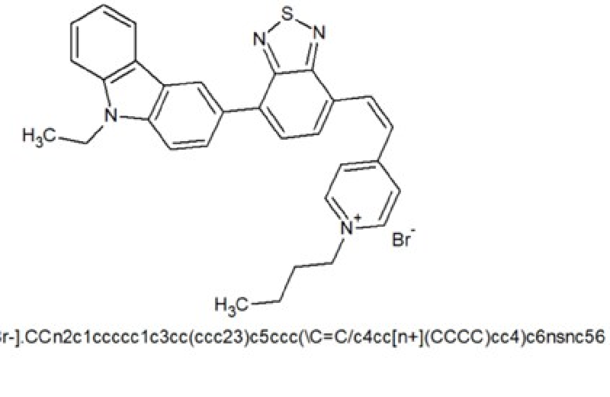

Figure S1. Procedure for generating SMILES notation using ACD/ChemSketch™.

2. The partition coefficient in n-octanol experimental determination of log P

According to published articles,[2] a certain amount of TZPI in octanol 2.0 mL was subjected to partition with octanol-saturated water 2.0 mL. The resulting mixture was stirred vigorously for 5 min., and centrifuged at 2,000 rpm for 5 min. The octanol layer was separated from water layer, and its fluorescence spectrum was recorded as FL-1. The above water layer was partitioned with water-saturated octanol 2.0 mL, and the octanol layer was separated after 5 min. vigorous stirring and 5 min. centrifuge at 2,000 rpm, and its spectrum was recorded as FL-2. As shown in Fig. S2, the emission peaks of the two spectra at 650 nm are 14820 and 410, respectively. The log P value was calculated by the fluorescence intensity ratio at 650 nm for the above two octanol extractions. (The above results are both obtained under the conditions of excitation wavelength of 480nm and excitation and emission slit width of 5nm).

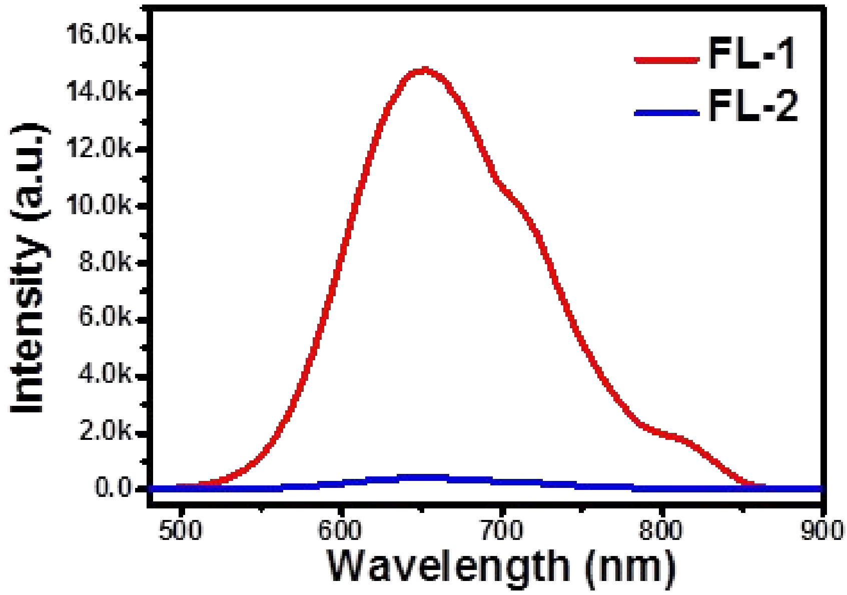

Figure S2. Fluorescence spectra of TZPI in the organic phase (FL-1) and in the water phase (FL-2).

Synthesis and characterization of TZPI

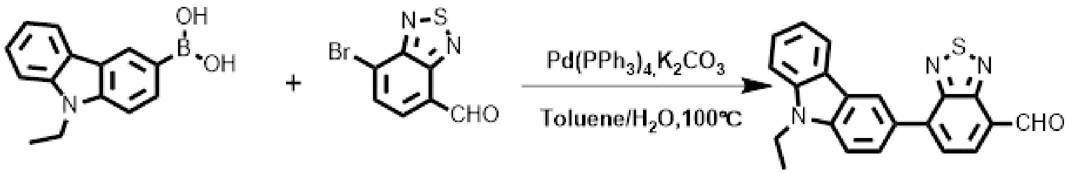

Scheme S1. Synthetic route of TZCHO.

der an atmosphere of nitrogen, to a mixture of (9-ethyl-9H- carbazol-3-yl) boronic acid (478 mg, 2 mmol), 7- bromobenzo [C] [1,2,5] thiadiazole-4-carbaldehyde (486 mg, 2 mmol) and Pd（PPh_3)$_4$ （84mg，0.073mmol）in toluene (20mL) was added a solution of K_2CO_3(2.0M, 1.5ml) in water, the reaction solution was refluxed overnight. Then the reaction mixture was poured into water and extracted with CH_2Cl_2 and dried over anhydrous Mg_2SO_4. The crude product was purified by column chromatography on silica gel（petroleum ether/ DCM = 1/2）to give a orange red powder of TZCHO (257 mg, 72% of the yield). ^{1}H NMR (600 MHz, DMSO-d6) δ 10.67 (s, 1H), 8.93 (d, J = 1.7 Hz, 1H), 8.40 (d, J = 7.3 Hz, 1H), 8.27 – 8.23 (m, 2H), 8.20 (d, J = 7.4 Hz, 1H), 7.82 (d, J = 8.6 Hz, 1H), 7.68 (d, J = 8.2 Hz, 1H), 7.52 (ddd, J = 8.2, 7.0, 1.2 Hz, 1H), 7.29 – 7.25 (m, 1H), 4.53 (q, J = 7.2 Hz, 2H), 1.38 (t, J = 7.1 Hz, 3H).

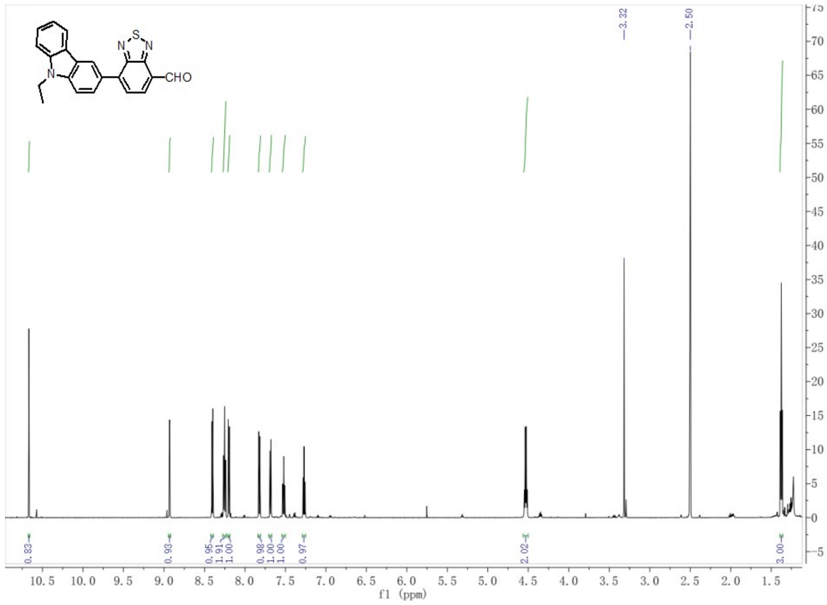

Figure S3. ^{1}HNMR spectrum of compound TZCHO.

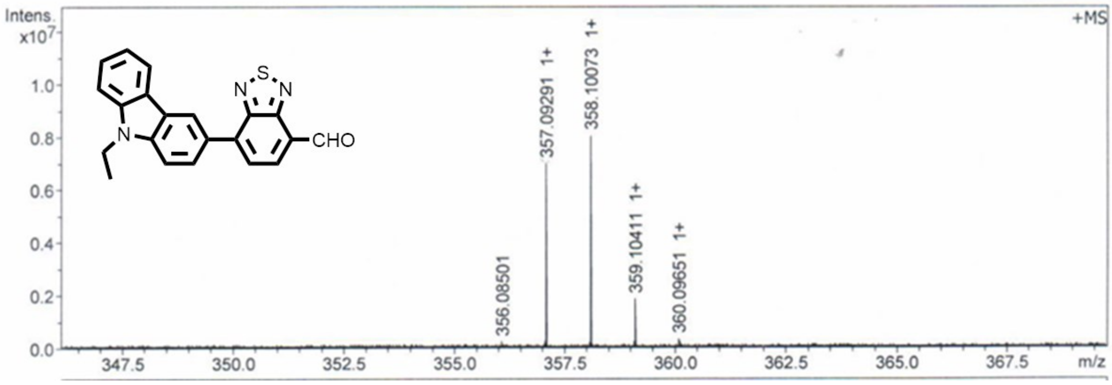

Figure S4. HR-MS (MALDI-MS) spectrum of compound，m/z = 357.

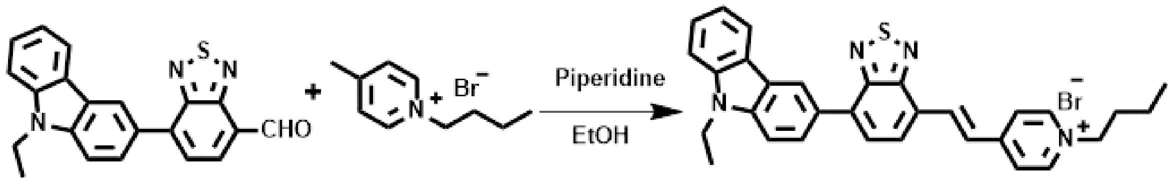

Scheme S2. Synthetic route of TZPI.

A solution of TZCHO (357 mg, 1mmol) and 1-Butyl-4-methylpyridinium Bromide (230 mg, 1 mmol) was refluxed under nitrogen in dry ethanol catalyzed by a few drops of piperidine overnight. After cooling to room temperature, the solvent was removed by evaporation under reduced pressure. The residue was purified by column chromatography on Al_2O_3 gel (methanol /DCM = 2/98 v/v) to give a red black powder of TZPI (302mg, 53% of yield). ^1H NMR (600 MHz, DMSO-d6) δ 9.02 (d, J = 6.4 Hz, 1H), 8.91 (d, J = 1.7 Hz, 1H), 8.48 – 8.34 (m, 4H), 8.28 – 8.19 (m, 3H), 8.15 (d, J = 7.4 Hz, 1H), 7.95 (s, 1H), 7.82 (d, J = 8.7 Hz, 1H), 7.69 (d, J = 8.2 Hz, 1H), 7.54 – 7.50 (m, 1H), 7.27 (t, J = 7.4 Hz, 1H), 4.54 (q, J = 7.3 Hz, 4H), 1.99 (dq, J = 12.9, 7.1 Hz, 2H), 1.36 (dt, J = 24.3, 7.3 Hz, 5H), 0.95 (t, J = 7.4 Hz, 3H).

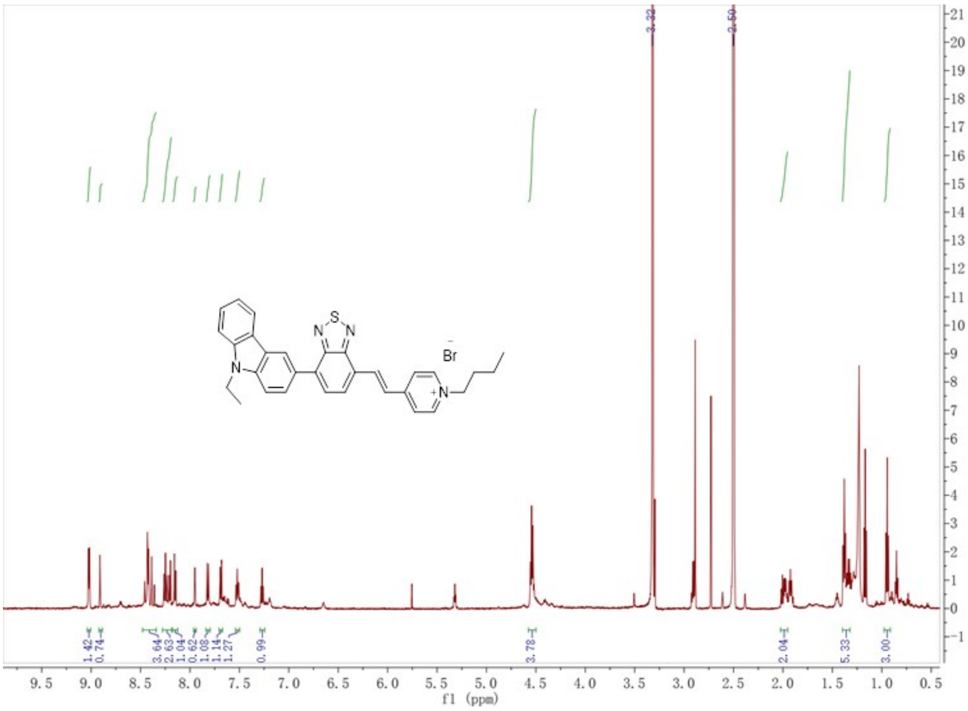

Figure S5. ^{1}HNMR spectrum of compound TZPI.

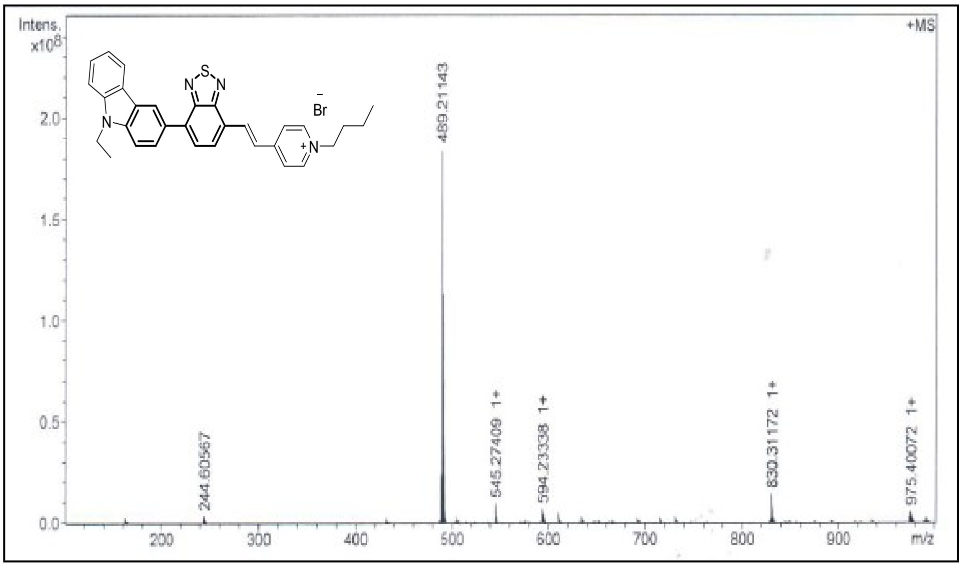

Figure S6. HR-MS (MALDI-MS) spectrum of compound TZPI， m/z(M-Br) = 489.

The method of fibrils formation and optical Measurement

Fibrils formation

Insulin amyloid fibrils were prepared according to the protocols described elsewhere.[3]

A stock solution containing Insulin (5 mg·mL^{-1}) and HCl (0.01 M, pH = 2) were prepared as shown in Scheme S3a.

A HCl (0.01 M, pH = 2) solution containing insulin (5 mg·mL^{-1}) and TPZI (10 μM) or ThT (10 μM) were prepared as shown in Scheme S3b.

The solutions were filtered through a 0.2 μm filter and then incubated in an orbital thermomixer with constant agitation at 600 rpm at 60℃ for 24 h. After heating, the solutions were cooled and then centrifuged (Jouan SA A14 centrifuge) at 3000 rpm for 3 min to remove any globular particulates. The supernatant liquid containing the fibrils was removed and stored at 4℃ until needed; this was used as the stock fibril solution. Fibril solutions were not used past 1 week.

Optical Measurement

The steady-state absorption spectra were measured on a Shimidazu UV-3600 UV-VIS-NIR spectrophotometer. The stationary fluorescence spectra and absolute fluorescence quantum yields were performed on a Horiba FluoroMax-4-NIR spectrophotometer equipped with an integrating sphere. The fluorescence lifetime was measured using an Edinburgh Instrument FLS980 Integrating sphere (FLS980 Series of Fluorescence Spectrometers).

In the study of using TZPI and ThT as an ex situ probe in scheme S3a, an aliquot of the insulin solution taken out from the incubation mixture at a defined time was diluted with Tris-HCl buffer, followed by the addition of the probe. The final concentrations of insulin, TPZI and ThT were 10 μg/mL, 20 nM and 20 nM, respectively.

In the study of using TZPI and ThT as an in situ probe in scheme S3b， TZPI or ThT was added to insulin solution prior to incubate at 60 °C with constant agitation at 600 rpm. Then an aliquot of the insulin solution with the probe taken out from the incubation mixture at a defined time was diluted with Tris-HCl buffer. The final

concentrations of insulin, TZPI and ThT were 10 μg/mL, 20 nM and 20 nM, respectively.

Under the condition of avoiding light, we shake the solution by hand for 10 seconds to mix well and make the fluorescence probe fully combine with the substance, and then the and then the spectra were measured on spectrophotometer.

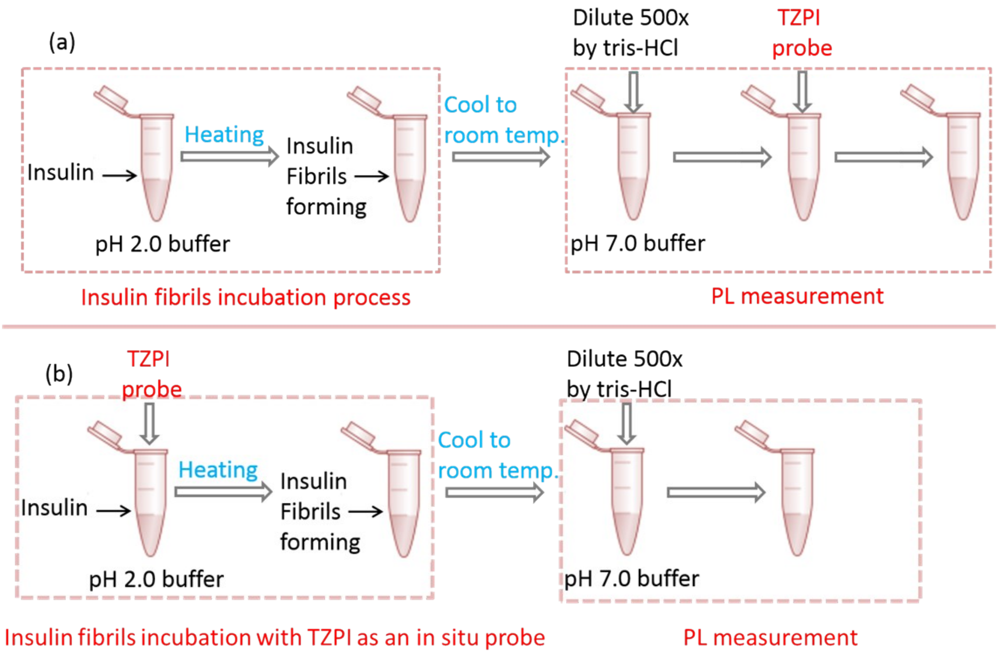

Scheme S3. Experimental procedures for the fibrils formation and optical measurement of insulin amyloid fibrils with TZPI **as an ex situ probe** (a) or **as an in situ probe** (b).

Transmission electron microscopy (TEM) experiment

In order to confirm the existence of fibrils, the solution of insulin was incubating for 24 h, which examined by TEM. The solution was diluted 20 times with 10 mM Tris–HCl buffer and a drop placed on a Formvar-coated grid. The resulting grid, after drying, was studied using a Hitachi H-7650 transmission electron microscopy, operating with an accelerating voltage of 80 kV.

The method of measuring the dissociation constant

The dissociation constants (K_d) of TZPI and ThT were derived by using a fixed concentration of the mature insulin fibrils with varying dye concentrations and fitted

using a one site binding equation. The resulting K_d for TZPI and ThT are 20 nM and 270 nM, respectively.

Two-photon fluorescence (TPF) microscopy experiment

TPA cross-sections were determined *via* a comparative method, by measuring the two-photon excited fluorescence (TPEF) using Rhodamine B as a reference. The fundamental of a mode-locked Ti: sapphire laser (690–850 nm, Tsunami) was focused into a quartz cuvette having an optical geometry, and detected with a liquid nitrogen cooled charge-coupled device (CCD) (SPEC-10-400 B/LbN, Roper Scientific) attached to a polychromator (Spectropro-550i, Acton).

Two-photon fluorescence imaging experiment

The two-photon imaging was investigated by a commercial microscope (TCS SP5 MP FCS, Leica Instruments). To explore the potential use of the TPZI for deep tissue TPF imaging, we investigated the imaging depth of TPZI in turbid tissue phantom. As shown in the schematic of our imaging setup (Scheme S4), the thickness between the two pieces of coverslips was adjusted by putting double-sided adhesive with different layers. And 1% intralipid solution was injected into the tunable gap of the two coverslips to form the simulative tissue. The scattering property of the simulative tissues was similar with the real tissue. A drop of TZPI/fibrils solution was dripped on the top coverslip. And an inverted two-photon microscope with a 20× long working objective lens (0.45 NA) was used to image the amyloid fibrils at different depths in the mock tissue.

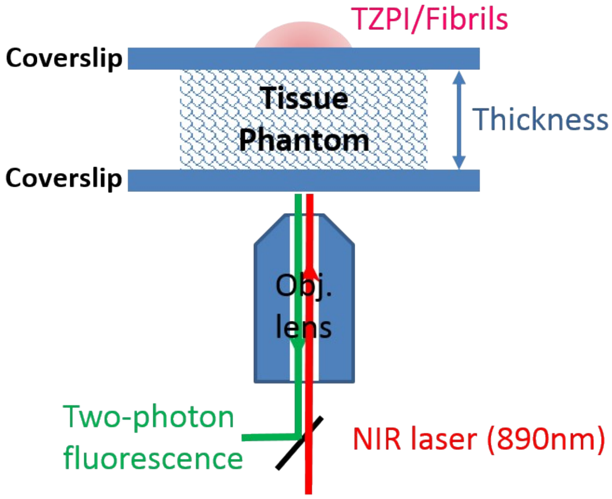

Scheme S4. Schematic of the setup used for TPFI of TZPI in tissue phantom with different thickness.

2. Supporting results and discussion

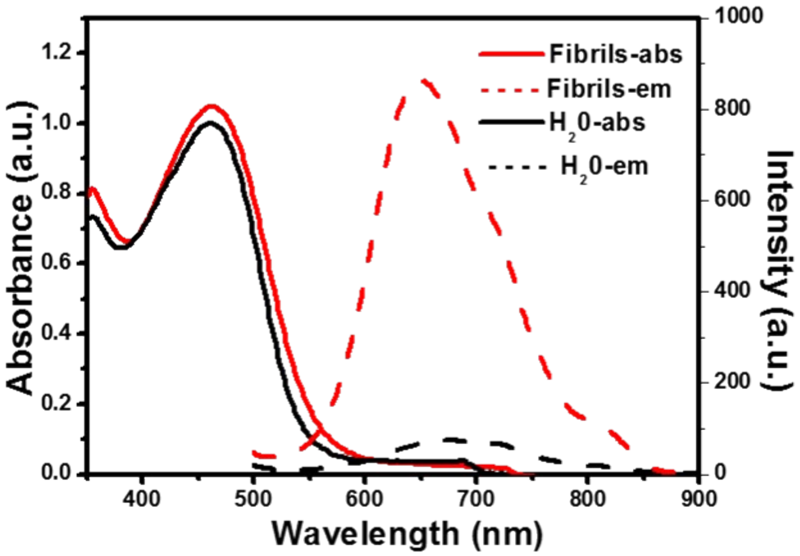

Figure S7. The absorption and emission spectra of TZPI dissolved in water (black) and mature fibrils solution (red).

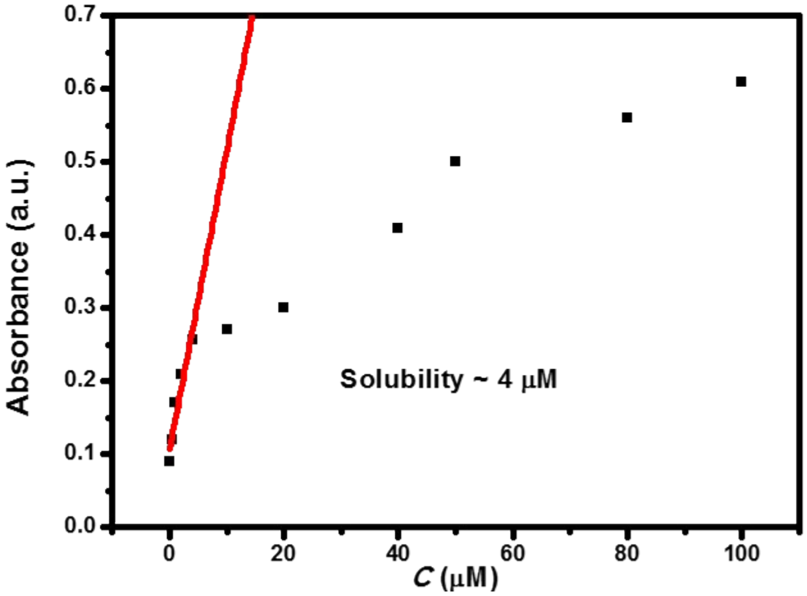

Figure S8. The plot of absorption intensity against probe concentration for TZPI in Tris-HCl buffer solution (10mM, pH 7.4).

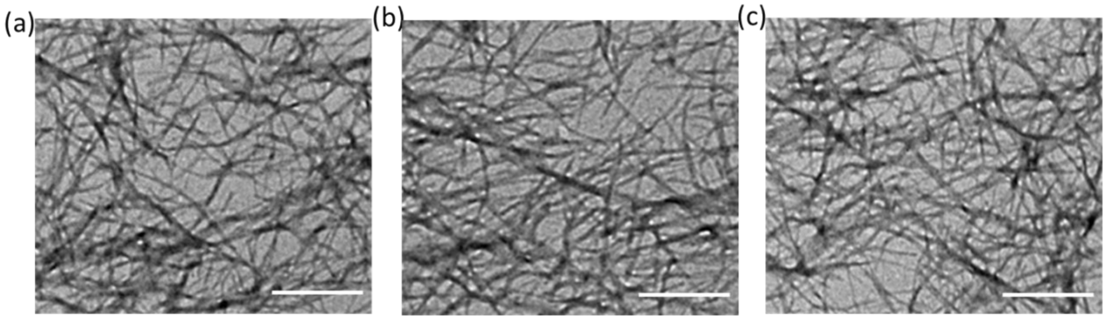

Figure S9. TEM image of insulin fibrils without probes (a), in the presence of TZPI (b) and in the presence of ThT (c). Scale bar: 500 nm. The fibrils have a length of several micrometres with an average width of 7-10 nm.

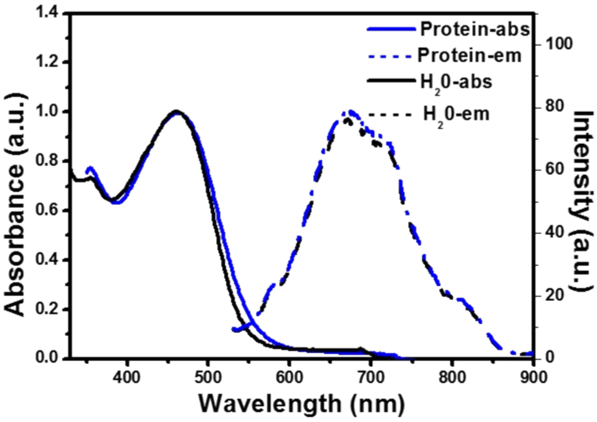

Figure S10. The absorption and emission spectra of TZPI dissolved in water (black) and native bovine insulin (blue).

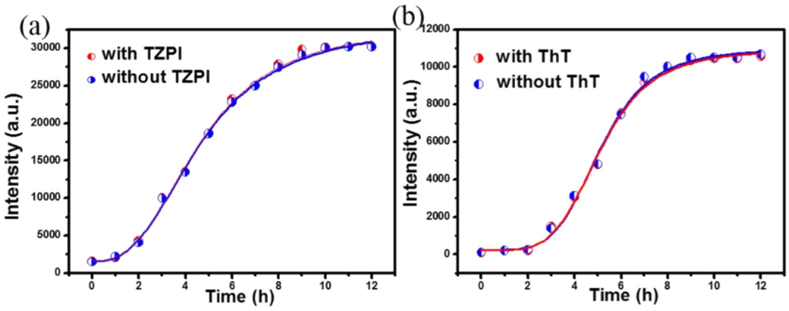

Figure S11. Integrated intensity of TZPI (a) and ThT (b) monitoring insulin (10 μg/mL) fibrillation, λ_{ex} =480 nm for TZPI, 410 nm for ThT. The concentrations of TZPI and ThT are 20 nM. The blue lines are TZPI and ThT as an ex situ probe. The red lines are TZPI and ThT as an in situ probe.

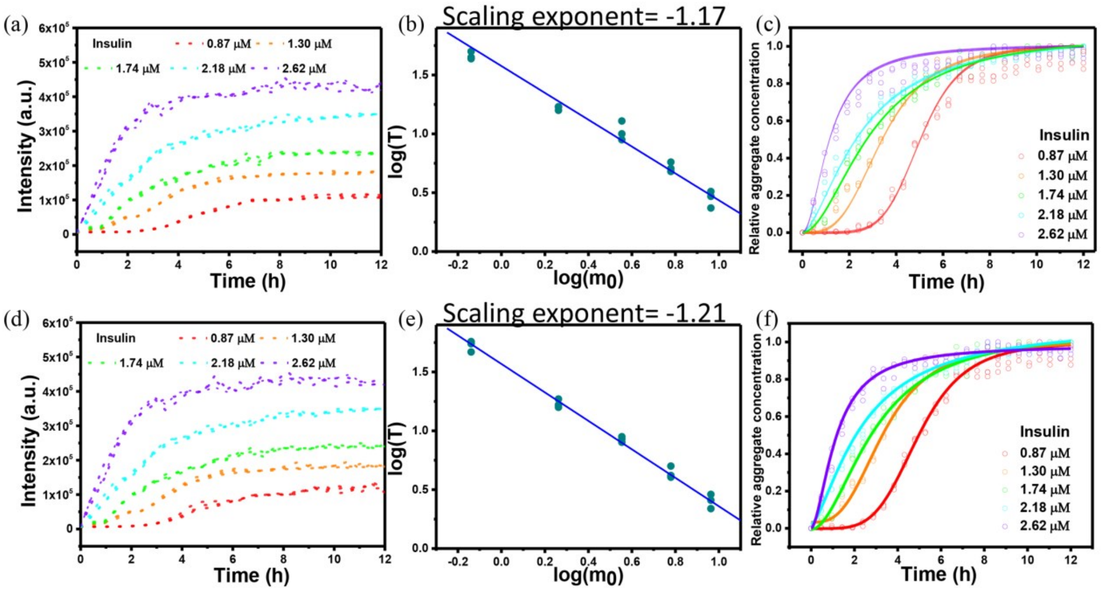

Figure S12. Experimental kinetics of insulin aggregation for 5 initial protein monomer concentrations (0.87-2.62 μM) and constant concentration of TPZ1 (20 nM) as an ex situ probe (a) and as an in situ probe (d).(b)(e) Power-law scaling of the time to half-completion with the initial monomer concentration. (c)(f)Global fits to the normalized experimental data.

- We carried out kinetic aggregation experiments by using TZPI as an in-situ or an ex-situ probe with a constant concentration of TZPI (20 nM) but varying concentrations of insulin (0.87-2.62 μM). As shown in Fig. S12(a) and Fig. S12 (d), the fluorescence data obtained can describe the kinetic process during the fibril formation.
- Theoretical analysis of aggregation kinetics was performed using the software AmyloFit (https://www.amylofit.ch.cam.ac.uk).The slope gave the scaling exponent γ was -1.17 and -1.21, respectively which might consistent with a dominant secondary nucleation pathway characterized by a monomer dependence of n_2= 2 and a contribution from primary nucleation with a reaction order of n_c= 2.
- As shown in Fig. S12(c) and Fig. S12(f), the data we fit based on the dominant secondary nucleation pathway were consistent with the experimental data over the full reaction time course. And the mean residual errors were both less than 0.03.

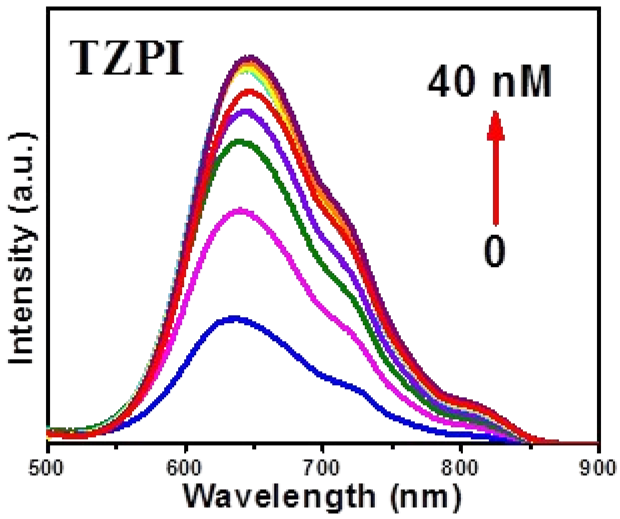

Figure S13. Fibrillary insulin in Tris-HCl (10 μg/mL) with different concentrations of TZPI solution (the excitation wavelength is 480 nm).

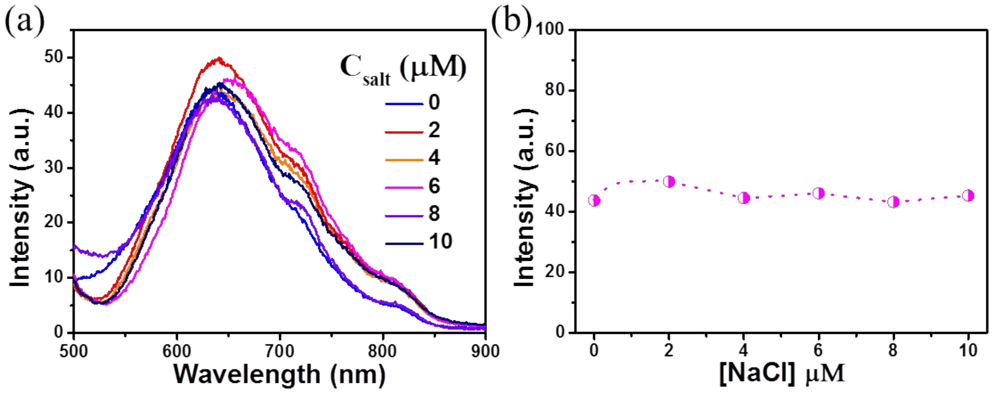

Figure S14. (a) The fluorescence intensity of TZPI in the presence of insulin fibrils with different salt concentrations. (b) Plot of the emission intensity for different salt concentrations.

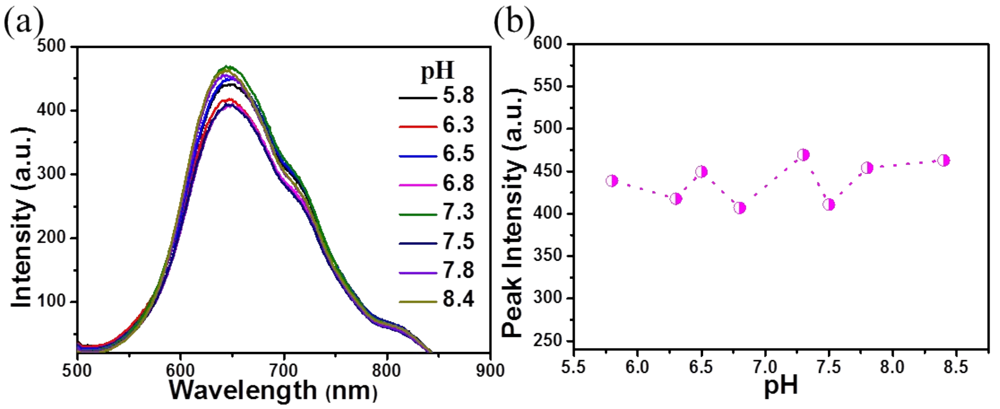

Figure S15. (a) The fluorescence intensity of TZPI in phosphate buffer with different pH values in the presence of insulin fibrils. (b) Plot of the emission intensity for different pH solution.

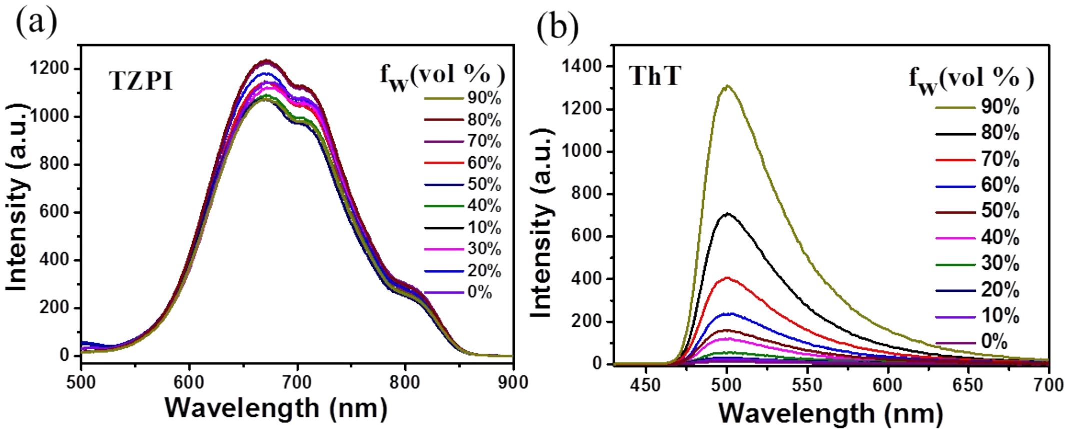

Figure S16. The emission spectra of TZPI (a) and ThT (b) in different ratios of glycerol: ethanol.

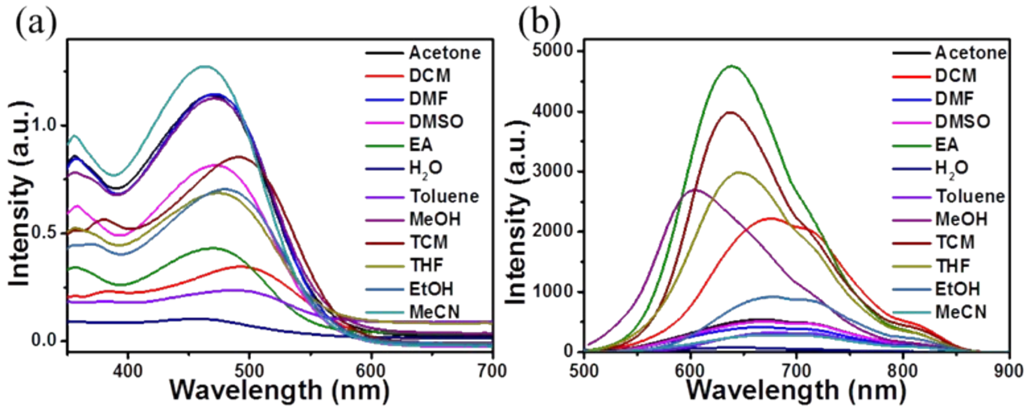

Figure S17. The absorption (a) and emission (b) spectra of TZPI in different solvents.

Solvent	ε	n	f(ε,n)	λ_a (nm)	λ_f (nm)	ν_a-ν_f (cm^{-1})	Φf (%)	τ (ns)	Kr ($\times 10^7 s^{-1}$)	Knr ($\times 10^8$ s^{-1})
**Toluene**	2.38	1.375	0.014	466	606	4958	40.23	4.05	9.93	1.48
**TCM**	4.81	1.445	0.148	470	634	5504	48.63	4.08	11.91	1.26
**THF**	7.58	1.407	0.210	475	644	5525	39.01	3.85	10.13	1.58
**Acetone**	20.7	1.359	0.284	477	668	5994	40.12	4.72	8.50	1.27
**EtOH**	24.3	1.361	0.288	475	668	6083	38.54	5.15	7.48	1.20
**Insulin**			0.297	460	648	6213	30.21	5.62	5.38	1.24
**H_2O**	80.4	1.333	0.320	462	660	6588	2.03	7.12	0.29	1.37

Table S1. Detailed photophysical data of TZPI in the different solvents and insulin.

Solvent	ε	*n*	*f(ε,n)*	λ_a (nm)	λ_f (nm)	$\nu_a - \nu_f$ (cm^{-1})
**Toluene**	2.38	1.375	0.014	414	490	3746
**TCM**	4.81	1.445	0.148	412	510	4664
**THF**	7.58	1.407	0.210	412	493	3988
**Acetone**	20.7	1.359	0.284	415	492	3771
**EtOH**	24.3	1.361	0.288	416	489	3589
**Insulin**			0.297	412	486	3696
**H_2O**	80.4	1.333	0.320	412	478	3351

Table S2. The Stokes displacement $(\nu_a - \nu_f)$ data of ThT in the different solvents and insulin.

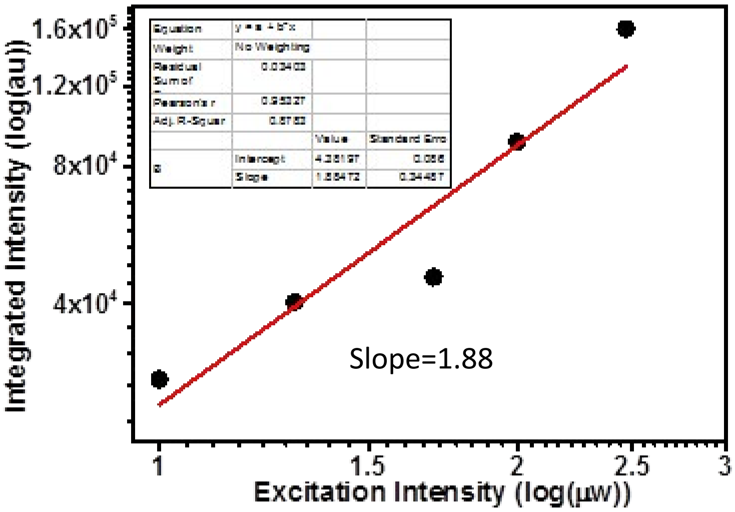

Figure S18. The peak intensity of two-photon excited fluorescence spectra versus laser intensity at 890 nm for TZPI solution in ethanol.

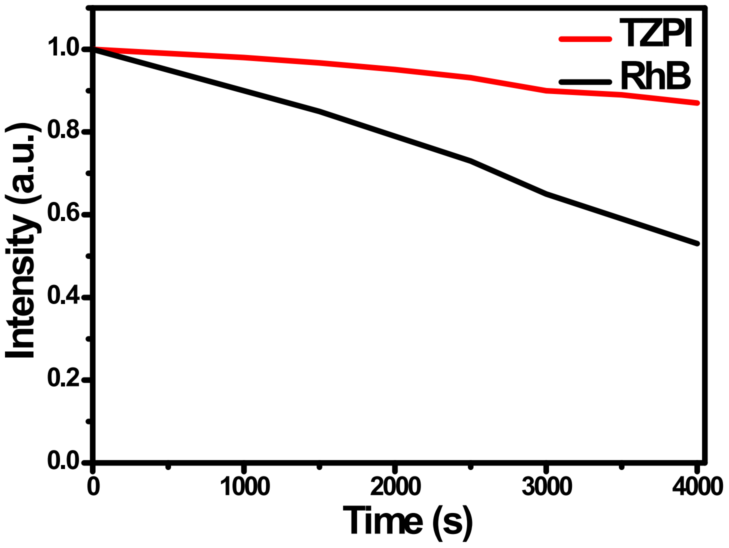

Figure S19. Photostability comparison between TZPI and RhB upon continuous excitation at 890 nm on a fixed spot for more than 4000 s.

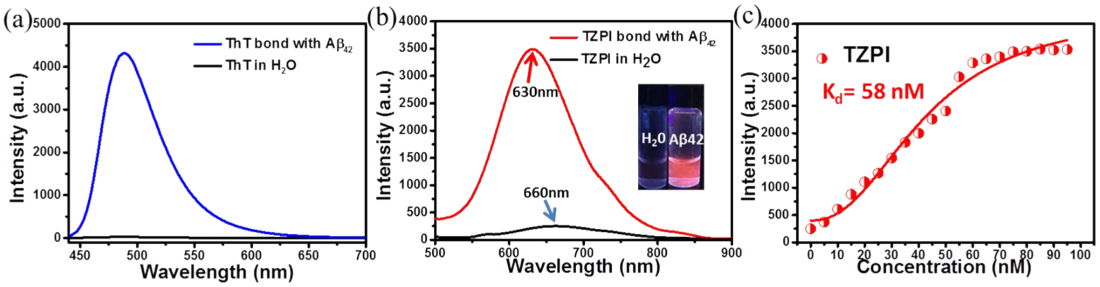

Figure S20. (a) Emission spectra of ThT (20 nM) dissolved in water (black) and mature fibrils solution (blue), respectively. λ_{ex} = 410 nm for ThT.(b) Emission spectra of TZPI (20 nM) dissolved in water (black) and mature fibrils solution (red), respectively. λ_{ex} = 480 nm for TZPI. Photographs of mixtures of TZPI with water and fibrillary forms of Aβ_{42} were taken under UV illumination. (c) The saturation binding of TZPI to fibrillar form of Aβ_{42}. The dissociation constants (K_d) of TZPI fitted using a one site binding equation. The resulting K_d for TZPI is 58 nM.

- We purchased Aβ_{42} from GL Biochem (Shanghai) Ltd and incubated the protein fibrils following the procedure reported in the literature[4] as follows: Aβ_{42} peptide

(0.25 mg) was dissolved in hexafluoro-2-propanol (HFIP, 0.2 mL) and incubated at room temperature for 1 h. HFIP was then removed by a flow of nitrogen and further dried by vacuum. HFIP-treated $A\beta_{42}$ was then dissolved in DMSO to a final concentration of 1 mM and diluted to 200 μM with 10 mM PBS buffer (pH= 7.4). The solution was incubated at 37 °C for 48 h with gentle and constant shaking.

- The formation of $A\beta_{42}$ fibrillary aggregates was confirmed by ThT assay. Upon addition of incubated amyloid fibrils in ThT, a significant emission intensity enhancement appears.
- A significant emission intensity (14-fold) enhancement of TZPI appears upon addition of incubated $A\beta_{42}$ fibrils. Meanwhile, the interaction of TZPI with the $A\beta_{42}$ amyloid fibrils leads to a bathochromic shift of the emission maximum from 660 to 630 nm.
- Furthermore，the K_d for TZPI/ $A\beta_{42}$ fibrils was derived by a fluorescence titration method and the value was found to be 58 nM, indicating a high binding affinity.

Reference:

1. J. Kujawski, M. K. Bernard, A. Janusz and W. Kuźma, *J. Chem. Educ*., 2011, 89, 64.
2. C. Ran, X. Xu, S. B. Raymond, B. J. Ferrara, K. Neal, B. J. Bacskai, Z. Medarova and A. Moore, *J. Am. Chem. Soc.*, 2009, 131, 15257.
3. C. C. Kitts, T. Beke-Somfai, B. Nordén, *Biochem.*, 2011, 50, 3451.
4. K. Rajasekhar, N. Narayanaswamy, N. A. Murugan, G. Kuang, H. Agren and T. Govindaraju, *Sci. Rep.*, 2016, **6**, 23668.
